# C3G Regulates STAT3, ERK, Adhesion Signaling, and Is Essential for Differentiation of Embryonic Stem Cells

**DOI:** 10.1007/s12015-021-10136-8

**Published:** 2021-02-23

**Authors:** Vijay V. Vishnu, Bh. Muralikrishna, Archana Verma, Sanjeev Chavan Nayak, Divya Tej Sowpati, Vegesna Radha, P. Chandra Shekar

**Affiliations:** CSIR-Centre for Cellular and Molecular Biology, Uppal Road, Hyderabad, 500 007 India

**Keywords:** C3G, RAPGEF1, Knock out, STAT3, ERK, Differentiation, Self-renewal, Transcriptome analysis, Adhesion, E14Tg2a mouse embryonic stem cells

## Abstract

C3G (RAPGEF1), engaged in multiple signaling pathways, is essential for the early development of the mouse. In this study, we have examined its role in mouse embryonic stem cell self-renewal and differentiation. C3G null cells generated by CRISPR mediated knock-in of a targeting vector exhibited enhanced clonogenicity and long-term self-renewal. They did not differentiate in response to LIF withdrawal when compared to the wild type ES cells and were defective for lineage commitment upon teratoma formation in vivo. Gene expression analysis of C3G KO cells showed misregulated expression of a large number of genes compared with WT cells. They express higher levels of self-renewal factors like KLF4 and ESRRB and show high STAT3 activity, and very low ERK activity compared to WT cells. Reintroduction of C3G expression in a KO line partially reverted expression of ESRRB, and KLF4, and ERK activity similar to that seen in WT cells. The expression of self-renewal factors was persistent for a longer time, and induction of lineage-specific markers was not seen when C3G KO cells were induced to form embryoid bodies. C3G KO cells showed poor adhesion and significantly reduced levels of pFAK, pPaxillin, and Integrin-β1, in addition to downregulation of the cluster of genes involved in cell adhesion, compared to WT cells. Our results show that C3G is essential for the regulation of STAT3, ERK, and adhesion signaling, to maintain pluripotency of mouse embryonic stem cells and enable their lineage commitment for differentiation.

**Supplementary Information:**

The online version contains supplementary material available at 10.1007/s12015-021-10136-8.

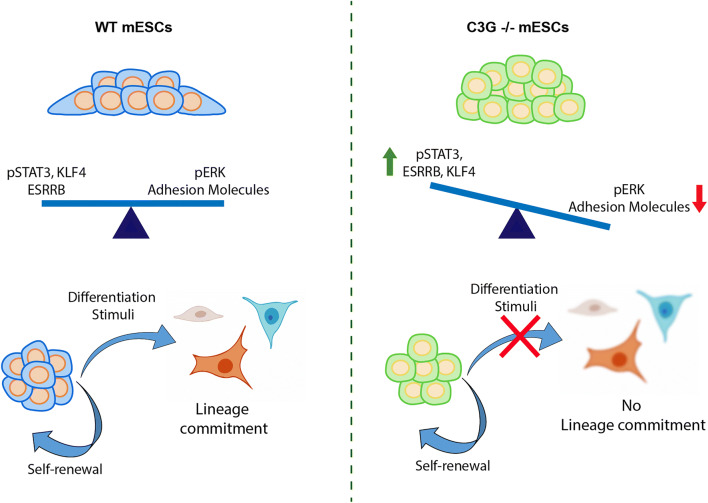

## Introduction

Pluripotent stem cells are characterized by a fine balance between self-renewal and their ability to differentiate to all cell types [1]. The interaction between the pluripotency factor network, signaling pathways, and epigenetic factors, establish and maintain pluripotency of stem cells [2]. Pluripotency factors, OCT4, SOX2, and NANOG activate the pluripotency network and early differentiation factors. The commitment of pluripotent stem cells to differentiation is restricted by LIF and BMP4 through activation of STAT3 and Id proteins respectively [3]. In addition, mechanical signals and cell adhesions originating from the physical interaction of pluripotent cells with their micro-environment affects cell fate decisions [4]. While the function of pluripotency factors, signaling pathways, and mechanical signals is appreciated in the regulation of pluripotency, the molecules and mechanisms controlling and connecting these pathways are poorly understood.

The guanine nucleotide exchange factor, C3G (RAPGEF1) is ubiquitously expressed and well conserved across vertebrates [5]. Mammalian C3G is a 140 kDa protein with a catalytic domain responsible for exchange factor activity in the C-terminus, a proline-rich protein interaction domain in the central region, and an N-terminal E-cadherin binding domain. It is engaged in response to growth factor, adhesion, and mechanical signals, and has effector functions dependent on its catalytic activity, as well as protein interactions. The small GTPases, Rap1, R-Ras, TC21, TC10, and MAPK pathways are regulated by C3G [6–8]. C3G and Rap1 play a major role in inside-out, and outside-in cell adhesion signaling [9, 10]. C3G is involved in signaling to gene expression and cytoskeletal reorganization, and therefore regulates diverse cellular responses like proliferation, adhesion, migration, survival, and differentiation. Mutations and deregulation of C3G expression are associated with a variety of disorders [11, 12].

C3G protein and transcripts are present in preimplantation embryos [13] and its expression is essential for embryogenesis. Mouse embryos lacking C3G die shortly after implantation [7]. Embryonic fibroblasts from KO mice expressing human C3G under Cre recombinase were defective in cell adhesion and spreading, and showed accelerated migration upon Cre activation. Mice expressing a hypomorphic allele of human C3G survived until later developmental stages (E14.5), with abnormalities in brain development [14, 15]. C3G is required for skeletal muscle and neuronal differentiation in vitro; [16, 17]. Expression of constitutively active C3G in hematopoietic stem and progenitor cells enhanced their differentiation and depleted progenitor cell population in the spleen [18].

The phenotype of mouse embryos lacking C3G suggested its role in regulating adhesion and lineage differentiation during post-implantation development. The function of C3G in mouse embryonic stem cells (mESCs) is not known, which prompted us to investigate its function in the maintenance of pluripotency. Using the mouse ES cell line, we show that the absence of C3G alters STAT3 and ERK signaling, expression of self-renewal factors, and adhesion properties, resulting in sustained self-renewal and resistance to differentiation.

## Material and Methods

### Embryonic Stem Cell Culture and Cell Line Generation

E14Tg2a mouse embryonic stem cells were cultured in ES cell media containing GMEM, 100 μM β-mercaptoethanol, supplemented with 1000 U/ml LIF, sodium pyruvate, non-essential amino acids, and 10% fetal calf serum in 0.1% gelatin-coated plates in a humidified atmosphere with 5% CO_2_ at 37 °C. For the generation of C3G null E14Tg2a cells, 1 million cells were nucleofected with knockout and HDR plasmid. pU6 GFP plasmid expressing Cas9 without the guide RNA was used as vector control (VC) for the experiment. After nucleofection cells were plated on a pregelatinized P100 plate in ESC media. The cells were selected with Puromycin for 7 days. Colonies were picked from the P100 Plate onto each well of a 96 well plate and were cultured until 70% confluency in replicas. Protein was isolated from one of the replica plates and knockouts were confirmed by western blotting. Full-length C3G cDNA was cloned into pFBEK plasmid under the control of the EF1α promotor (pFBEK-C3G). For the reintroduction of C3G into null cells, two million C3G knockout cells were nucleofected with 2 μg of pFBEK C3G plasmid, and cells were seeded on to a 60 mm culture dish. After 24 h, C3G expression was confirmed in the lysates, by western blotting and was used for examining other proteins.

### Colony Formation and LIF Withdrawal Assay

Colony formation assay was done with Alkaline Phosphatase Detection Kit (Sigma- SCR004) as described previously [19]. For LIF withdrawal assay, cells were cultured in varying concentrations of LIF from 0 to 1000 U/L followed by alkaline phosphatase staining. The percentage of undifferentiated, mixed, and differentiated colonies were calculated and the data was plotted using Prism 8.0, *n* = 3.  ± SD of the experiment was plotted as error bar. The images were captured in bright field, 20x magnification using an Olympus IX3 microscope.

### Western Blot Analysis

Whole-cell protein was prepared from the cells using RIPA buffer. The proteins were probed with appropriate primary and secondary antibodies (Supplementary Table-1) and the blots were developed using ChemiDoc MP (BioRad). Densitometry analysis was done using Image-J. Actin was used as a control for the normalization of protein in all the samples. The data were plotted as mean normalized protein level relative to WT. ± SD was plotted as error bar. Data from at least three independent experiments were plotted and significance was calculated with unpaired t-test using Prism 8.0.

### qPCR

Knock out and control cells were cultured as mentioned above. Total RNA from the cells was isolated using RNAiso Plus (TaKaRa Cat. #9108/9109) as per manufacturer’s protocol. DNA was removed from the samples by incubating the samples with Turbo DNAse at 37 °C for 30 min and followed by heat inactivation of the enzyme at 65 °C for 5 min. First-strand cDNA synthesis was performed using Takara First-strand cDNA synthesis Kit (#6110A). Briefly, 1 μg of RNA was taken from each sample and was mixed with 1 μl of 50 μM oligoDT primer, 1 μl of 10 mM dNTP mix, and 5 μl DEPC water. The mixture was incubated at 65 °C for 5 min. Second strand synthesis was done by adding 1 μl of 10x RT buffer, 2 μl of 25 mM MgCl_2_, 1 μl 0.1 M DTT, 0.5 μl of RNase Out, and 0.5 μl of the superscript enzyme to the above reaction mixture. The tubes were further incubated at 50 °C for 50 min followed by heat inactivation at 85 °C for 5 min. RNA from the samples were degraded by adding 1 μl of RNase H and incubating at 37 °C for 20 min.

qPCR was done by using Power SYBR™ Green PCR Master Mix (Thermo Fisher Scientific) as per the manufacturer’s protocol with appropriate primers (Supplementary Table-2) in a 384 well PCR plate. The plate was run on QuantStudio 5 Real-Time PCR System (Thermo Fisher Scientific). The reaction conditions were as follows: 95 °C for 5 min followed by 40 cycles of 95 °C for 10 s, 60 °C for 30 s, and 72 °C for 30 s. The data were analyzed using Graph Pad Prism 8.0.

### RNA Sequencing and Data Analysis

The RNA seq was performed using NovaSeq6000. The quality assessment of Paired-End sequence reads of all the samples was done using FastQC [20]. The Alignment and identification of transcripts were done by STAR [21] using Ensembl Mouse genome (GRCm38). Comparative gene expression analysis was done between wild type versus C3G gene knockout cells. All analyses were performed within the R environment and most plots were produced with the ggplot2 package [22] and Enhanced Volcano [23]. Differential gene expression analysis was performed using DESeq2 [24]. Moderated estimation of fold change and dispersion for RNA-seq data was done with DESeq2.

Gene Ontology analysis and KEGG enrichment analysis were performed to explore the biological pathways that involve differentially expressed genes. GO analysis was conducted by the enrichGO function of cluster Profiler [25]. The *p* value of the GO term and the false discovery rate (FDR) of the p value (q-value) were calculated to find out the most relevant GO term for the genes with differential expression. Pathway enrichment analysis was performed using the KEGG Mapper.

### EB Differentiation Assay

C3G knock out cells and control cells were grown in hanging drop culture for 48 h and EB’s were transferred to –LIF media to facilitate differentiation. EB’s were collected every three days starting from day 0 till 12 days, and RNA was isolated using trizol. qPCR was done subsequently to determine the levels of pluripotency and differentiation markers in WT and knock out cell line. The data were normalized to the RNA level of Day 0 WT cells.

### Teratoma Formation and Histological Analysis

WT and C3G null cells, were trypsinized and were washed twice in PBS, and were resuspended in PBS at a final concentration of 4 × 10^4^ cells per microliter. 100 μl of the cell suspension was injected subcutaneously to the hind leg of a 6-week-old SCID mouse (5 animals in each set). After 21 days, the animals were sacrificed, teratomas resected and tumor volume was measured. For histological analysis, teratomas were fixed in buffered formalin for 72 h and embedded in paraffin wax blocks. 5 μm sections were taken and H&E and Masson’s trichrome staining were carried out according to standard procedures. The images were captured in a bright field, 10X magnification using an Olympus IX3 microscope.

### Cell Adhesion Assay

1000 cells of C3G knockout clone D3 and WT were seeded onto each well of a 6-well plate. The cells were grown for 7 days. On the 7th day, the cells were washed twice with PBS and were incubated with 200 μl of 0.05% trypsin solution for one to five minutes. The trypsin was inactivated by adding serum-containing media after every minute of incubation. After incubation, the media was removed and cells were washed with PBS. The attached colonies were fixed with 70% ethanol for 5 min and were stained with 1% methylene blue in an aqueous solution for 5 min. After incubation, the dye was removed and the plate was washed with PBS. The number of colonies in each condition was counted using OpenCFU software and the graph was plotted using Prism 8.0.

### Quantification and Statistical Analysis

All the experiments were repeated at least twice and statistical significance was calculated with a minimum experiment number of 3. All error bars represented in the figures are shown as mean ± SD. The statistical significance between groups was determined with an unpaired Students t-test. For all the statistical tests, the 0.05 confidence level was considered statistically significant. In all the figures * denotes *p* < 0.05, **denotes *p* < 0.01 and ***denotes *p* < 0.001. Prism 8.0 was used for plotting all the graphs and statistical analysis. OpenCFU software was used for colony counting in trypsin assay.

## Results

### Loss of C3G Enhances Self-Renewal of mES Cells

**Fig. 1:**
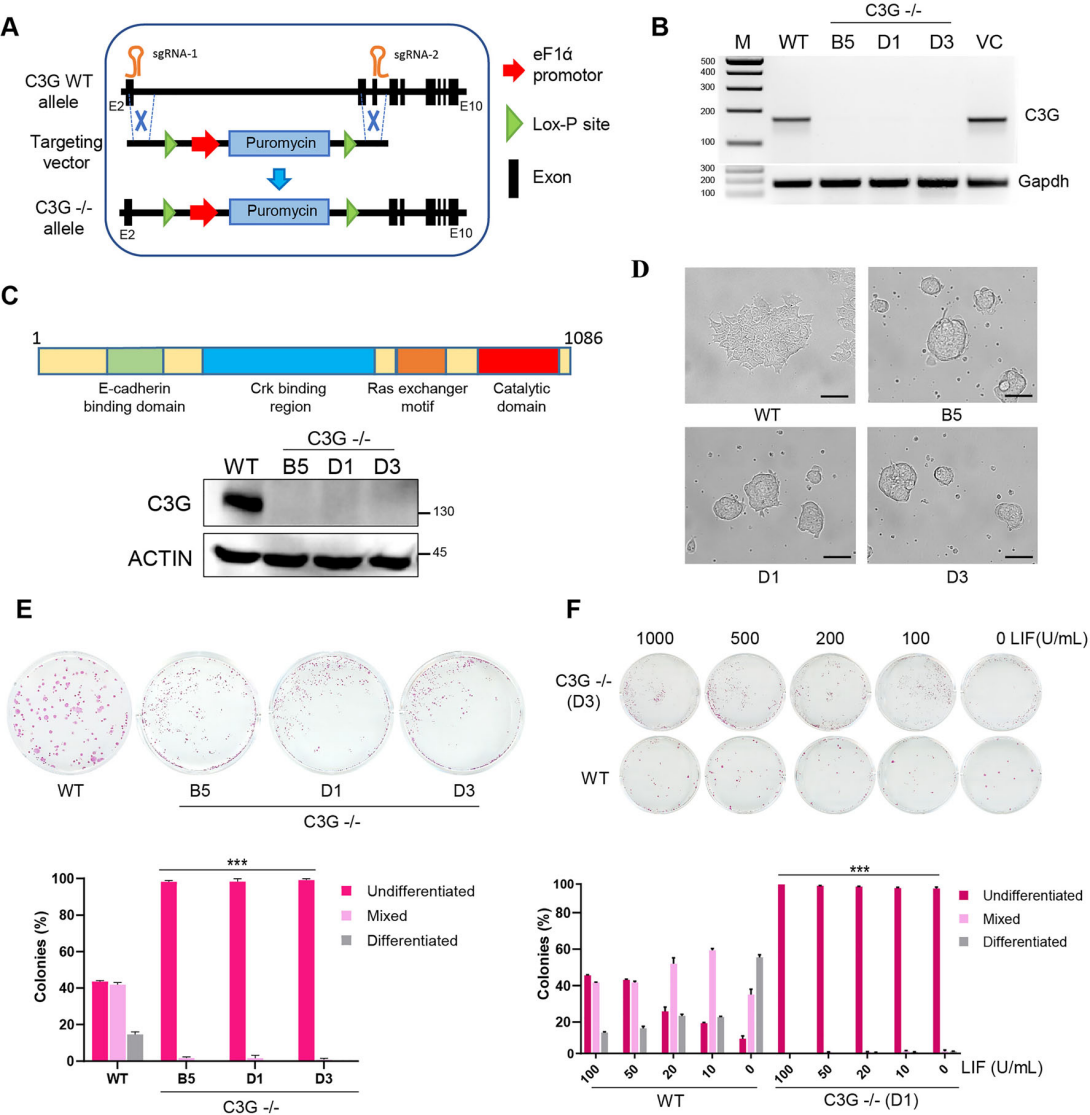
C3G null ES cells exhibit enhanced self-renewal. **a** Schematic showing the 5′ region of mouse C3G gene indicating exons and guide RNA positions. EF1α-Puromycin cassette was knocked-in to replace exons 2–4 of the C3G gene. **b** RT-PCR analysis of C3G transcripts in three independent knockout clones (B5, D1 & D3), WT, and vector control (VC) using N-terminal primers. **c** Schematic representation of C3G protein domains and western blot showing C3G expression in WT and knockout clones. Actin was used as a loading control. **d** Bright-field images of representative colony morphology of WT cells and C3G KO clones. Scale bar, 40 μm. **e** Clonogenic assay of WT and KO cells grown for 7 days. The bar diagram shows the quantification of colonies with the indicated morphology. Data were averaged from three independent experiments carried out in duplicates, ***P < 0.001. **f** Clonogenic assay of WT and C3G KO clone D1 grown in the absence and presence of indicated LIF concentrations. Quantification of colony type is shown in the bar diagram. ***P < 0.001; *n* = 3.

We generated C3G knockout mESC lines using CRISPR mediated knock-in of a targeting vector with Puromycin selection cassette in E14Tg2a cells. A pair of sgRNAs targeting 3’end of intron-1 and intron 4 were used to replace a 7 kb region encompassing exons 2–4 (Fig. 1a and S1A). Puromycin resistant individual colonies were propagated and screened for C3G expression. E14Tg2a parent cells (WT), and clones selected after the introduction of the vectors without guide RNAs (VC), were used as controls. To exclude the possibilities of clonal variation, we chose three C3G KO clones for further experiments. C3G transcripts were not detected using primers corresponding to the deleted exons (Fig. 1b). However, transcripts were seen in the KO clones when primers corresponding to C-terminal exons were used (Fig. S1b and S1c), indicating the production of truncated transcripts despite the disruption of the N-terminal exons. No polypeptides corresponding to full-length C3G, (Fig. 1c) or any truncated protein were seen when examined using a monoclonal antibody that detects sequences in the C-terminal half of C3G [26] (Figs. S1b and S1d).

Morphologically, C3G knock-out cells formed compact round and raised colonies with no sign of differentiated cells unlike wild type, and vector control cells which formed typically spread-out colonies (Fig. 1d). Unlike WT cells which formed undifferentiated, mixed, and differentiated colonies, null cells did not form alkaline phosphatase negative differentiated colonies when assayed for colony formation (Fig. 1e and S1e). The null cells predominately (>98%) formed tightly packed undifferentiated colonies, with high alkaline phosphatase activity, suggesting that C3G null cells possess high clonal self-renewal capability. Clonogenic assay was performed using one of the null clones (D1) in the presence of varying LIF concentrations. As expected, the proportion of the mixed and differentiated colonies steadily increase with a decrease in LIF concentration in WT cells. However, C3G nulls cells did not show differentiated colonies even in the absence of LIF (Fig. 1f). These results suggested that C3G knock out cells have persistent self-renewal ability, and do not require LIF for self-renewal, and fail to differentiate upon withdrawal of LIF. Despite persistent self-renewal, C3G null ESCs exhibited a slow rate of proliferation as determined by MTT assay (Fig. S1f). Cell cycle analysis by FACS showed accumulation of cells in the G1 phase compared to controls, suggesting that delayed G1/S transition is responsible for their slower proliferation (Fig. S1g). Together our data suggest that the loss of C3G leads to LIF independent self-renewal and delayed G1/S transition in ES cells.

### ESCs Lacking C3G Fail to Differentiate In Vitro and In Vivo

**Fig. 2:**
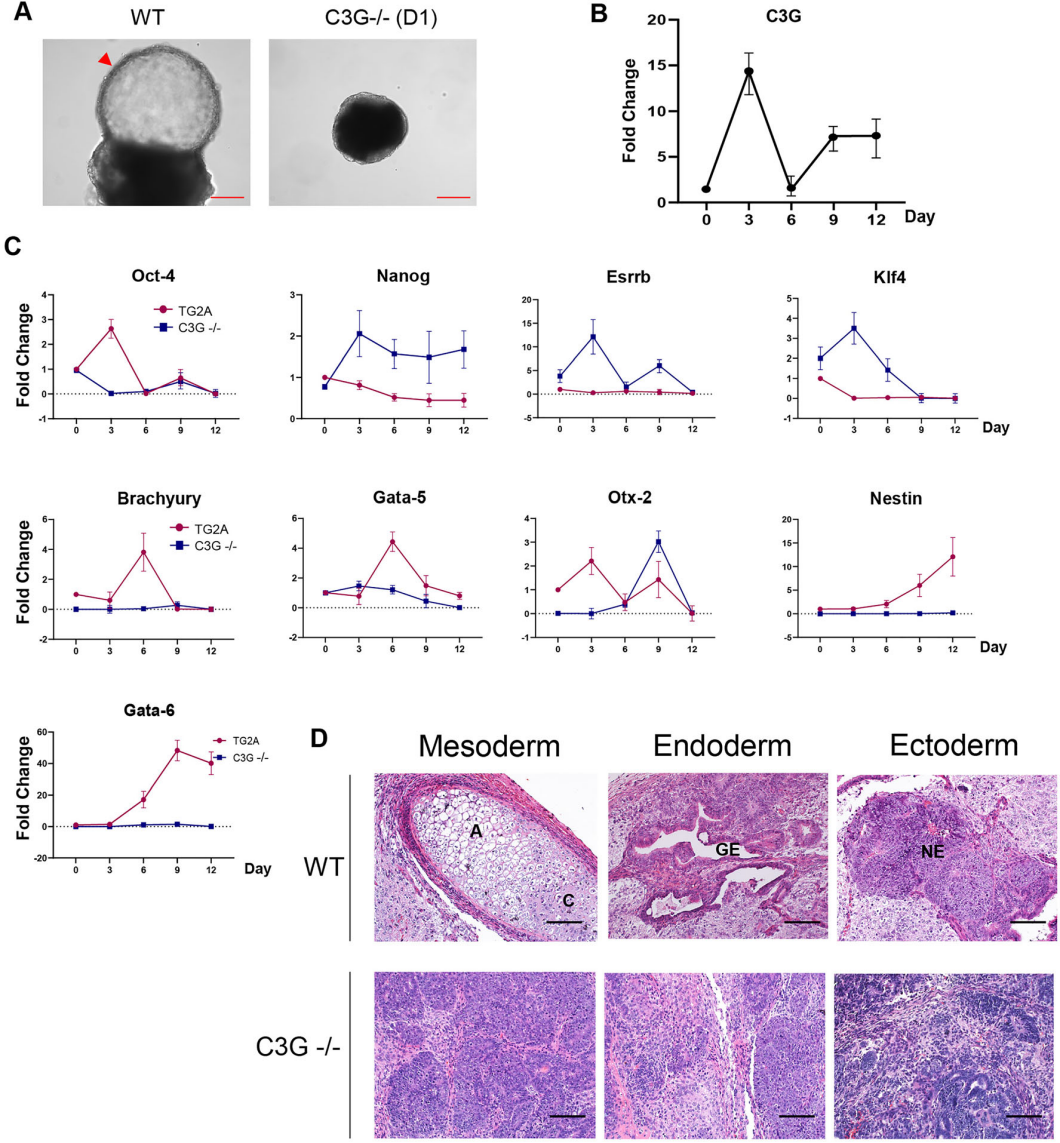
ESCs lacking C3G fail to differentiate in vitro and in vivo. **a** Representative images (bright-field) of 12 days old EBs formed by WT and C3G null clone (D1). Arrowhead indicates the cystic cavity. Scale bar, 200 μm. **b** qPCR analysis of C3G expression during EB differentiation in WT cells **c** qPCR analysis of expression of the indicated genes during EB differentiation, RNA samples were collected from Day 0 to Day 12, and GAPDH was used as an internal control. n = 3. **d** H&E stained histological sections of 21-day old teratomas. Images show representative sections taken from different areas of the teratoma. A-Adipocytes; C-Cartilage; G-Gut Endoderm; NE-neuroepithelium. Scale bar, 100 μm.

Since the loss of C3G caused persistent self-renewal of the mESCs, we asked if the loss of C3G disrupts the balance between self-renewal and differentiation. The cells were allowed to spontaneously differentiate into all the germ layers in suspension as embryoid bodies (EBs) for 12 days. C3G null cells assembled into simple embryoid bodies, which were relatively smaller, and lacked cystic cavity when grown for 12 days, compared to those formed by WT cells, suggesting impaired differentiation (Fig. 2a). Since C3G is essential for early post-implantation development, and embryoid bodies can model many aspects of post-implantation development, we analyzed C3G expression during embryoid body differentiation [27]. C3G transcripts were induced several folds during the first three days of EB differentiation in WT cells. Though its levels decreased by day 6, they increased again and were higher than levels, before LIF withdrawal (Fig. 2b). This suggests the induction of C3G correlates with the differentiation of ES cells.

To understand the nature of the differentiation defect, we analyzed the expression of pluripotency factors and genes that induce the three germ layers during EB differentiation in WT and C3G null EBs. *Oct4* expression was initially induced and later repressed as differentiation continues. However, in the C3G null EBs, *Oct4* was repressed during differentiation suggesting that Oct4 did not inhibit differentiation. *Nanog *expression was not repressed in C3G null EBs upon differentiation and remained persistently high till day12, compared to the repression seen in WT EBs. The naive pluripotency factors *Esrrb* and *Klf4*, which were expressed at high levels in null cells were further upregulated early during EB differentiation. Overall, it was observed that *Nanog, Essrb,* and *Klf4* expression was induced during EB differentiation of C3G null cells, unlike the repression seen in EBs formed by WT cells (Fig. 2c). Since the overexpression of *Nanog, Essrb*, and *Klf4* enhances self-renewal and represses differentiation, we analyzed the transcription factors responsible for the differentiation of the three germ layers during EB growth. Unlike in WT EBs, the germ layer markers, *Brachyury, Gata5, Gata6, Otx2*, and *Nestin* were not induced in null EBs or expressed very late (*Otx2*) in differentiation (Fig. 2c). Interestingly, C3G levels were induced ahead of many of the lineage determinants during the EB differentiation in WT cells. These results suggest an essential role for C3G in the induction of factors required for differentiation of ES cells to all germ layers.

Consistent with EB differentiation, C3G null cells formed smaller teratomas with lower average volume at three weeks, relative to WT cells (Fig. S2a). Teratomas formed by null cells had to be resected with host tissue as it invaded into host tissue. H&E staining showed extensive angiogenesis and tissue invasion relative to WT teratomas (Fig. S2b and S2c). Unlike the WT, which showed tissue belonging to the three germ layers, C3G null teratomas formed a relatively homogenous mass of cells that resembled sarcomatous tissue with little differentiation and no tissue-level organization upon H&E and Masson’s trichome staining (Fig. 2d and S2d). Together, our data show that C3G is essential for repression of pluripotency factors during differentiation, and transition of ESCs from self-renewal to differentiation. These data show that C3G is essential for repression of pluripotency factors during differentiation, for the transition of ESCs from self-renewal to differentiation.

### C3G Is Essential for the Maintenance of the Pluripotency Factor Network

**Fig. 3:**
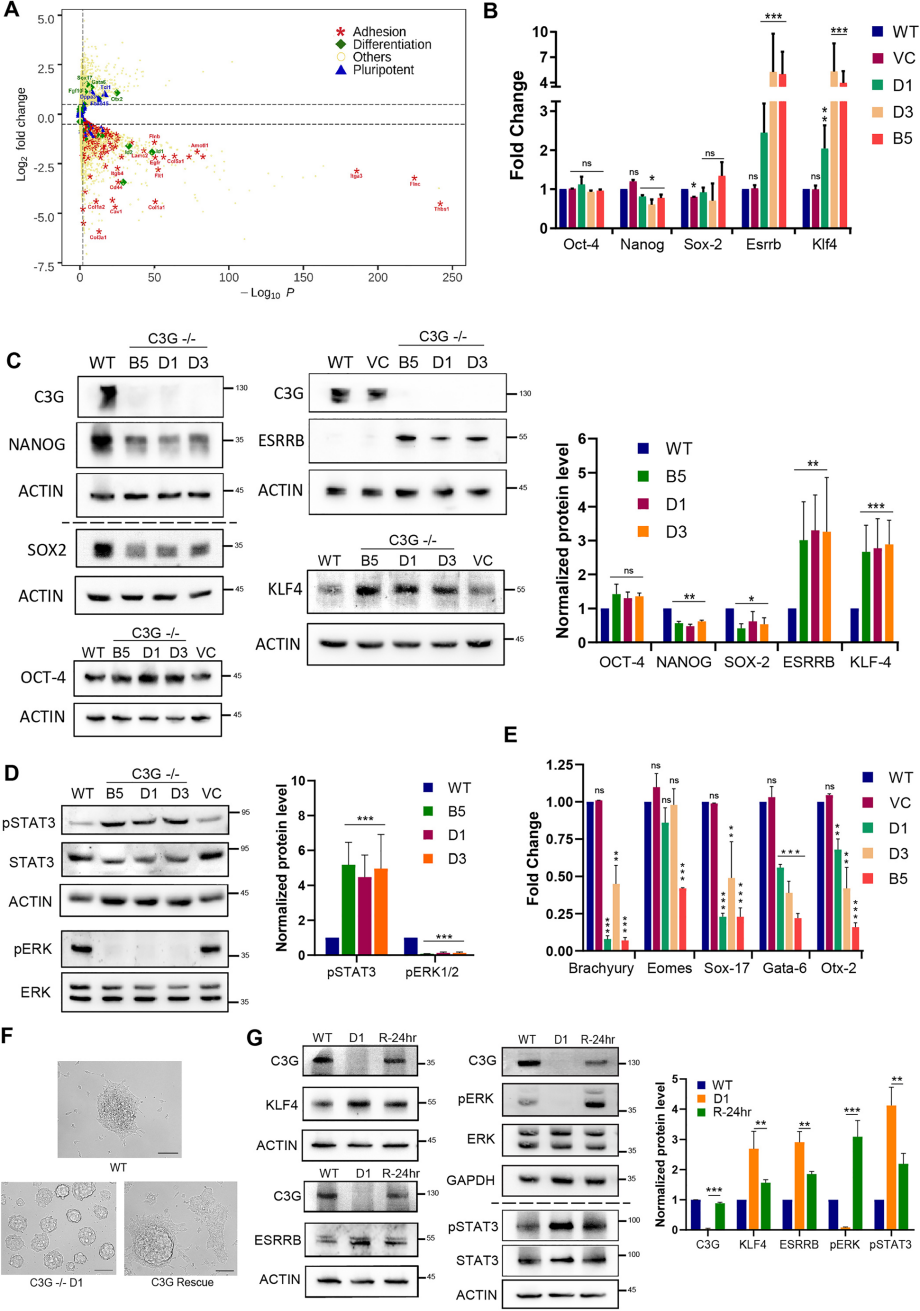
C3G is essential for the maintenance of the pluripotency factor network. **a** Volcano plot showing changes in the global expression of genes in C3G knockout cells compared to WT cells. Dotted lines indicate the significance of *p* < 0.05. Significantly altered expression of genes involved in pluripotency, differentiation, and adhesion are highlighted. **b** qPCR analysis of indicated pluripotency factors in vector control (VC) and C3G KO cells relative to WT cells.*P < 0.05, **P < 0.01, ***P < 0.001; n = 3 **c** Western blots showing expression of the indicated proteins in WT, VC, and C3G KO clones. Quantification averaged from three independent experiments relative to levels seen in WT is represented in the bar diagram. ACTIN was used as a loading control. **d** Western blot showing pSTAT3, and pERK levels. Quantification of levels normalized relative to respective total proteins is shown in the bar diagram, *p* < 0.001; n = 3. **e** Quantification of differential expression of indicated differentiation factors determined by qPCR. *P < 0.05, **P < 0.01, ***P < 0.001, n = 3. **f** Colony morphology of WT, KO clone D1, and D1 clone upon transient expression of C3G (R-24). **g** Western blots showing expression levels of indicated proteins in KO clone (D1) upon the rescue of C3G expression. Quantification relative to expression in WT cells is shown in the bar diagram. *P < 0.05, **P < 0.01 ***P < 0.001; n = 3.

The pluripotency factor network in ES cells is regulated by multiple mechanisms to maintain pluripotency. Deregulation of the network results in loss of self-renewal or potential to differentiate. The consequence of C3G loss on gene expression was examined by transcriptome analysis of three independent WT, and two independent C3G null samples. Differential expression analysis identified significant upregulation of 2657 genes, and downregulation of 3140 genes in C3G null cells. Interestingly, many of the pluripotency genes did not show a significant difference in transcript levels relative to WT (Fig. 3a). The expression of multiple genes involved in cell adhesion, PI3K/AKT signaling, and cancer was significantly reduced, and those involved in metabolic and ribosome function were upregulated in null cells (Fig. 3a, S4a and S4b).

The LIF independent self-renewal of C3G null ES cells is similar to the self-renewal observed in ES cells with overexpression of pluripotency genes like *Nanog* or *Esrrb* [28, 29]. Analysis of core pluripotency factors by q-PCR did not show significant changes in *Oct4* and *Sox2*. *Nanog* transcripts were found to be relatively lower than the WT, and the naïve state factors - *Esrrb* and *Klf4* were significantly higher in C3G null cells (Fig. 3b). NANOG and SOX2 protein expression were 30–40% lower in the null cells, and OCT4 did not change significantly (Fig. 3c). Immunostaining showed heterogeneous expression of NANOG in both WT and C3G null cells (Fig. S3). Consistent with their transcript levels, ESRRB and KLF4 proteins were significantly higher in C3G null cells (Fig. 3c). Although *Esrrb* was considered to be a NANOG target, and show concurrent expression [30], more recent studies have shown that neither *Esrrb* nor *Klf4* are direct targets of Nanog [31].

LIF /STAT3 signaling pathway primarily induces *Esrrb* and *Klf4* [32]. Since C3G null cells could self-renew in the absence of LIF, we analyzed STAT3 activation in these cells. pY705-STAT3 levels were 2–4 fold higher in C3G null cells (Fig. 3d) compared to the levels in WT. We analyzed the expression of some of the early lineage genes to evaluate the consequence of high STAT3 activity. As expected, many of the early markers of the three germ layers viz. *Brachyury, Eomes, Sox17, Gata6* & *Otx2* were repressed in C3G null cells. (Fig. 3e). Our data suggested that C3G null mESCs have constitutively high STAT3 activity, which desensitizes it to external LIF signal and perturbs the pluripotency gene network. The elevated STAT3 may be responsible for high expression of its downstream genes, *Esrrb* and *Klf4*, promoting continuous self–renewal. Surprisingly, the expression of another downstream gene, *Nanog* was low and could be due to the feedback repression process. Our data suggest that C3G is essential for the regulation of STAT3 activity in mESCs, which maintains a balanced pluripotency gene network.

In response to various stimuli, C3G engages the MAPK pathway in a cell type-dependent manner [33, 34]. C3G participates in FGFR1 signaling to regulate MAPKs [35] and our RNA seq analysis identified significant differences in expression of *Fgf4* and *Fgfr1*, in addition to other Fgf family genes in C3G null cells. FGF4 is a major stimulus for ERK activation, essential for lineage commitment in mESCs, and inhibition of ERK activity is pivotal for ESC differentiation [36, 37]. pERK1/2 level in C3G null cells was significantly lower than in WT (Fig. 3d). The near absence of ERK1/2 activity in cells lacking C3G was akin to a situation mimicking MEK1/2 inhibition preventing priming and differentiation of mESCs. We therefore compared the global changes in transcripts seen in C3G KO cells with changes seen in murine ESCs treated with MEK inhibitor, from GEO database [38]. Interestingly, we found multiple genes and pathways commonly up or down regulated between cells lacking C3G expression, and those treated with MEK inhibitor (Fig. S4c & S4d).

To confirm that the molecular changes seen in C3G null cells were indeed due to loss of C3G, we reintroduced C3G in one of the null clones, under transient conditions. 24 h post-transfection, C3G was expressed at near similar levels to that seen in WT cells. The cells showed signs of differentiation and loss of C3G null ES cell colony morphology (Fig. 3f). These cells showed a decrease in expression of ESRRB, KLF4, pSTAT3 and an increase in pERK level compared to the null cells (Fig. 3g), indicating that C3G expression can rescue some of the differentiation defects in the null cells.

### C3G Is Essential for the Regulation of Cell Adhesion

**Fig. 4:**
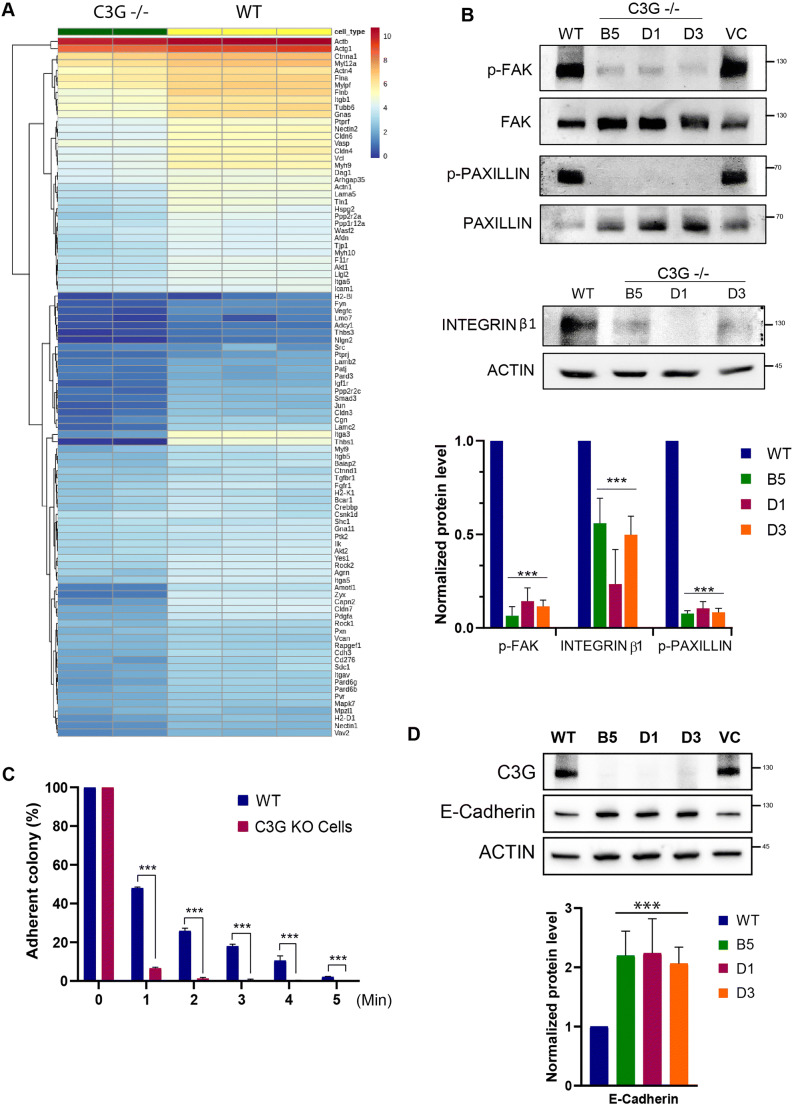
C3G is essential for the regulation of cell adhesion. **a** Heat map showing differential expression of cell adhesion-related genes in C3G KO cells compared to WT cells. **b** Western blots showing levels of indicated proteins. Actin was used as a loading control. Relative levels averaged from three experiments are shown in the bar diagram. pFAK and pPaxillin were normalized against corresponding total proteins.***p < 0.001, n = 3. **c** C3G knock out cells show reduced adhesion, Quantification of adherent colonies at indicated time points upon trypsin treatment is shown in bar diagram (see also S4C), ***p < 0.001, n = 3. **d** Western blots showing E-cadherin protein levels in the indicated cells. Actin was used as loading control and quantitation of the expression averaged from multiple experiments is shown in the bar diagram p < 0.001, *n* = 4.

C3G regulates cell adhesion in multiple cell types to regulate development [39–41]. Transcriptome analysis and gene ontology analysis of C3G null cells showed general repression of genes involved in cell adhesion processes, ERK signaling and negative regulation of STAT3 pathways (Fig. 4a, S4a and S4b). The downregulation of cell adhesion genes was consistent with the colony morphology of C3G null cells which were spherical and raised (Fig. 1d). *Integrin β1* (ITGB1) transcript and protein were significantly low in C3G null ES cells (Fig. 4a and b). Integrins regulate the self-renewal of ESCs and blocking of integrins inhibits differentiation [42]. Cell adhesion molecules and the ECM regulate self-renewal, pluripotency, and cell fate choice [42, 43]. Focal adhesions (FAs) containing integrins are the contact points of cells to the extracellular matrix. Focal adhesion kinase (FAK) and Paxillin are activated by phosphorylation at the FAs (pY705FAK, and pY118Paxillin), and C3G null cells showed low levels of pFAK and pPaxillin (Fig. 4b). The low levels of pFAK and pPaxillin are consistent with the downregulation of ITGB1 in null cells. Weak adhesion to the substratum enables self-renewal of mouse ES cells [44], and integrin engagement can trigger differentiation [42, 45]. We evaluated if C3G null cells differed in their ability to attach to the substratum. Over 90% of the C3G null cells/colonies could be detached from the culture dishes within one minute when treated with Trypsin unlike the WT cells, which took 3–4 min (Fig. 4c and S5a).

Since C3G KO cells grew as compact colonies, we examined cell-cell junction formation by staining for E-cadherin. In WT as well as KO cells, E-cadherin was present at cell junctions (Fig. S5b). Total cellular levels of E-cadherin were higher in the KO cells (Fig. 4d) suggesting formation of tighter cell junctions by KO cells. These results show that C3G KO cells adhere poorly to the substratum, but show stronger cell-cell adhesion. These results suggest that C3G is essential for the expression of ITGB1, the formation of FAs, and activation of ERK1/2 in ES cells to maintain them in a pluripotent state.

## Discussion

This study demonstrates that C3G is essential for enabling mESC differentiation, but is dispensable for their self-renewal. C3G null cells exhibit constitutively high STAT3 activity, and very low ERK activity, consistent with their self-renewal even in the presence of differentiation stimuli [36]. In mESCs, C3G may be inhibiting kinases, or activating phosphatases that regulate STAT3 tyrosine phosphorylation [46, 47]. Functional interaction of C3G with tyrosine kinases and phosphatases that regulate STAT3 have been described [48, 49].

Loss of C3G promotes LIF independent self-renewal of ES cells similar to the loss of many lineage commitment factors [36, 50, 51]. LIF signaling maintains pluripotency by activating STAT3, and induction of the naïve pluripotency factors Klf4 and Esrrb. [30, 32, 52, 53]. Surprisingly, NANOG and SOX2 levels are lower in C3G null cells despite elevated levels of pSTAT3. NANOG functions are dispensable in presence of high ESRRB [30, 32]; hence marginally lower NANOG levels seem to have no tangible impact on the self-renewal of C3G null cells. Partial reversion in pERK, ESRRB, and KLF4 levels upon expression of C3G, further confirmed that the molecular changes seen in C3G null cells are indeed due to the absence of C3G.

Persistent expression of pluripotency genes hampers temporal expression of developmental genes and reinforces pluripotency network to promote self-renewal even in absence of LIF. Induction of C3G during early stages of EB differentiation, failure of post-implantation development of C3G null embryos [7], and failure to form cysts in C3G embryoid bodies suggest that C3G expression is crucial for exit from a pluripotent state to enable gastrulation and commitment to three germ layers. We propose that expression of C3G in the early stage of differentiation is essential for suppression of pSTAT3, KLF4, and ESRRB, to permit timely expression of differentiation factors and lineage commitment.

While C3G null cells continue to self-renew, they proliferate slower, with an elongated G1 phase, compared to WT cells, indicating that absence of C3G compromises proliferation as well as differentiation. In mESCs, the undifferentiated state can persist, even with an elongated G1 phase resulting from deregulated expression of cell cycle genes [54]. Loss of C3G impacts the expression of cell cycle genes in epithelial cells and myocytes [16, 17, 55], and could be playing a similar role in mESCs.

In MEFs, C3G regulates adhesion-dependent on its catalytic activity [7]. Though C3G deficient MEFs did not differ in MAPK activation induced by cell adhesion or EGF stimulation, activation of Drosophila C3G resulted in cell fate changes, and over-proliferation during development mediated by MAPK [56]. In hematopoietic progenitors, Rap1 activation significantly enhanced ERK activation [57]. Also, EGF induced plasma membrane ERK activation, and cell morphology changes involve Rap1 [58]. C3G dependent signaling through activation of Rap is required for integrin-dependent adhesion [59]. Integrin and FAK engagement can also activate ERK1/2 in many cell types [60]. The shared pattern of gene expression changes seen in C3G KO cells, and mouse ESCs treated with MEK inhibitors provides additional evidence that C3G could be regulating ERK activation triggered by growth factors, and integrin engagement.

How C3G engages various downstream effectors in mESCs resulting in the observed molecular changes, and the cause-effect relationships await further investigation. Several properties of C3G may be responsible for its function in mESCs. C3G localizes to the mother centriole and regulates centriole division and primary cilia length, which can impact cell fate decisions [55]. In addition to its role in cytoplasmic signaling, it undergoes regulated nucleo-cytoplasmic exchange and regulates chromatin modifications, and mRNA splicing, important for temporal changes in gene expression [61, 62]. Together, our results suggest that C3G plays multifunctional roles in mESC differentiation. On one hand, it participates in the restriction of differentiation inhibitory pathways involving STAT3, Klf4, and Esrrb to make ESCs permissive for differentiation. On the other hand, it activates ERK and FAs, to enable lineage commitment.

In summary, our study reveals the essential role of C3G in the modulation of growth factor, cytokine, and adhesion signaling pathways to restrict naïve pluripotency factors, and activate gene regulatory networks, to regulate the delicate balance between self-renewal and differentiation of pluripotent stem cells.

## Supplementary Information

ESM 1 (DOCX 19 kb)

ESM 2 (JPEG 1.55 mb)

ESM 3 (JPEG 3.54 mb)

ESM 4 (JPEG 746 kb)

ESM 5 (JPEG 1.43 mb)

ESM 6 (JPEG 1.12 mb)

## Declarations

### Availability of Data and Material

All the data will be freely available and the material will be freely available after the material transfer agreement.

### Code Availability

The RNA-seq analysis reported in this paper is deposited in Gene Expression Omnibus under accession number GEO: GSE159595.
