# Supplementary material for: C3G Regulates STAT3, ERK, Adhesion Signaling, and Is Essential for Differentiation of Embryonic Stem Cells
Source: Stem Cell Rev Rep. 2021 Feb 23;17(4):1465–77. doi: 10.1007/s12015-021-10136-8 (PMC8372029; doi:10.1007/s12015-021-10136-8)
Supplement: Supplementary file 1 — (DOCX 19 kb) [file 12015_2021_10136_MOESM1_ESM.docx]

**Supplemental Information**

**SUPPLEMENTAL EXPERIMENTAL PROCEDURES**

**Immunostaining**

Cells were fixed with 4% paraformaldehyde for 10 min and cells were washed with PBS containing .1% triton X100 (PBST). The cells were blocked with 4% BSA in PBST for 1hr followed by appropriate primary antibody incubation overnight at 4°C. Cells are washed twice in PBST and was incubated with corresponding secondary antibodies at room temperature for 1hr. After two washes in PBST the cells were mounted in Vectashield and images were taken using Zeiss Axio Observer microscope.

**Supplementary Table-1**

| **REAGENT or RESOURCE** | **SOURCE** | **IDENTIFIER** |  |
| --- | --- | --- | --- |
| **Cell Lines** | | | |
| E14Tg2a |  |  |  |
| E14Tg2a C3G-/- | This paper |  |  |
| **Plasmids** | | | |
| C3G CRISPR/Cas9 KO Plasmid (m) | Santa Cruz | sc-430750 |  |
| C3G HDR Plasmid (m) | Santa Cruz | sc-430750-HDR |  |
| pFBEK C3G | This paper |  |  |
| **Antibodies** | | | |
| Rabbit anti-NANOG | CST | Cat# 8822, RRID:AB_11217637 |  |
| Rabbit anti-Oct-4 | CST | Cat# 2840, RRID:AB_2167691 |  |
| Rabbit anti-ESRRB | INVITROGEN | Cat# PA1-315, RRID:AB_2100260 |  |
| Goat anti-Klf-4 | R&D Systems | Cat# AF3158, RRID:AB_2130245 |  |
| Rabbit anti-Phospho Stat-3 (tyr705) | CST | Cat# 9131, RRID:AB_331586 |  |
| Mouse anti-Stat-3 | CST | Cat# 9139, RRID:AB_331757 |  |
| Rabbit anti-Sox-2 | CST | Cat# 23064, RRID:AB_2714146 |  |
| Phospho Paxillin | CST | 69363 |  |
| Paxillin | CST | Cat# 2542, RRID:AB_10693603 |  |
| Rabbit anti-Phospho-p44/42 MAPK(Erk1/2) | CST | Cat# 9101, RRID:AB_331646 |  |
| P44/42 MAPK (Erk1/2) | CST | Cat# 9107, RRID:AB_10695739 |  |
| Rabbit anti-FAK | CST | Cat# 3285, RRID:AB_2269034 |  |
| Rabbit anti-Phospho-FAK (Tyr397) | CST | Cat# 3283, RRID:AB_2173659 |  |
| Rabbit anti E-Cadherin | CST | Cat# 3195, RRID:AB_2291471 |  |
| Rabbit anti-Integrin β-1 | Chemicon | Cat# AB1952P, RRID:AB_571025 |  |
| Mouse anti-C3G | In house antibody | 3F6 |  |
| Mouse anti-β-Actin | Millipore | Cat# MAB1501, RRID:AB_2223041 |  |
| Mouse anti-GAPDH | Millipore | Cat# MAB374, RRID:AB_2107445 |  |
| Sheep anti-Mouse IgG HRP | GE-Amersham | Cat# NA931, RRID:AB_772210 |  |
| Donkey anti-Rabbit IgG HRP | GE-Amersham | Cat# NA9340-1ml, RRID:AB_772191 |  |
| Anti-Goat IgG HRP | SantaCruz | Cat# sc-2020, RRID:AB_631728 |  |
| Donkey anti-Rabbit IgG Alexa Fluor Plus 647 | INVITROGEN | Cat# A32795, RRID:AB_2762835 |  |
| Donkey anti-Mouse IgG Alexa Fluor Plus 488 | INVITROGEN | Cat# A32766, RRID:AB_2762823 |  |
| **Softwares** | | | |
| Prism 8.0.0 | Graphpad | www.graphpad.com |  |
| ImageJ | Schneider, et al., 2012 | https://downloads.imagej.net/fiji/ |  |
| Zen | Carl Zeiss Microsystems | https://www.zeiss.com/microscopy/us/products/microscope-software/zen.html |  |
| OpenCFU | Geissmann., 2013 | http://opencfu.sourceforge.net/ |  |
| **Reagents** | | |  |
| Leukocyte Alkaline Phosphatase Kit | Sigma | 86R-1KT |  |
| P3 Primary Cell 4D-NucleofectorTM X Kit L | Lonza | V4XP-3024 |  |
| RNAiso Plus | TaKaRa | Cat # 9108/9109 |  |
| First-strand cDNA synthesis Kit | TaKaRa | Cat # 6110A |  |
| Gelatin | Sigma | Cat # G1890 |  |
| GMEM | Sigma | Cat # G6148 |  |
| NEAA | Gibco | Cat # 11140-050 |  |
| Sodium pyruvate | Sigma | Cat # P5280 |  |
| β-mercaptoethanol | Sigma | Cat # M3148 |  |
| FBS | Gibco | Cat #10270-106 |  |
| VECTASHIELD® Antifade Mounting Media | Vector Laboratories | Cat # H-1200 |  |

**Supplementary Table-2**

| **Gene** | **Primer Sequence** |
| --- | --- |
| **Oct-4** | GTGGAGGAAGCCGACAACAATGA |
|  | CAAGCTGATTGGCGATGTGAG |
| **Nanog** | TGGTCCCCACAGTTTGCCTAGTTC |
|  | CAGGTCTTCAGAGGAAGGGCGA |
| **Klf-4** | GTGCAGCTTGCAGCAGTAAC |
|  | AGCGAGTTGGAAAGGATAAAGTC |
| **Esrrb** | TCTTCCCAGCTCCCACAGTA |
|  | CCCCATGCAAGCTTCGTAGT |
| **Sox-2** | TTTTCTAGTCGGCATCACCG |
|  | ACAAGAGAATTGGGAGGGGT |
| **Nestin** | GAAGGTGGGCAGCAACTGGCA |
|  | AGCTTCAGCTTGGGGTCAGGAA |
| **Gata-5** | CGTGGCACCCACTCGCATGC |
|  | GCTGTGAGCGAACGGGAAGGT |
| **Gata-6** | TTGCTCCGGTAACAGCAGTG |
|  | GTGGTCGCTTGTGTAGAAGGA |
| **GAPDH** | CAACGGCACAGTCAAGGCCGA |
|  | CCCTTCAAGTGGGCCCCGG |
| **Otx-2** | GGCCTCACTTTGTTCTGACC |
|  | CTTCATGAGGGAAGAGGTGG |
| **Brachyury (T)** | GTGGTGTGTAATGTGCAGGG |
|  | ATAACGCCAGCCCACCTACT |
| **Eomes** | ACCAAAACACGGATATCACCCAGC |
|  | GGGACAATCTGATGGGATCTAGGGG |
| **Sox-17** | CGCACGGAATTCGAACAGTA |
|  | GTCAAATGTCGGGGTAGTTG |
| **C3G C-Terminal** | GTCAAATGTCGGGGTAGTTG |
|  | CCACCGCTTGGAGAAGTT |
| **C3G N-Terminal** | AAAGCAGACTCTCAGCGTTCT |
|  | TCTCAGGAATCTTCGACACCTC |
|  |  |
