# Supplementary figures and images for: C3G Regulates STAT3, ERK, Adhesion Signaling, and Is Essential for Differentiation of Embryonic Stem Cells
Source: Stem Cell Rev Rep. 2021 Feb 23;17(4):1465–77. doi: 10.1007/s12015-021-10136-8 (PMC8372029; doi:10.1007/s12015-021-10136-8)

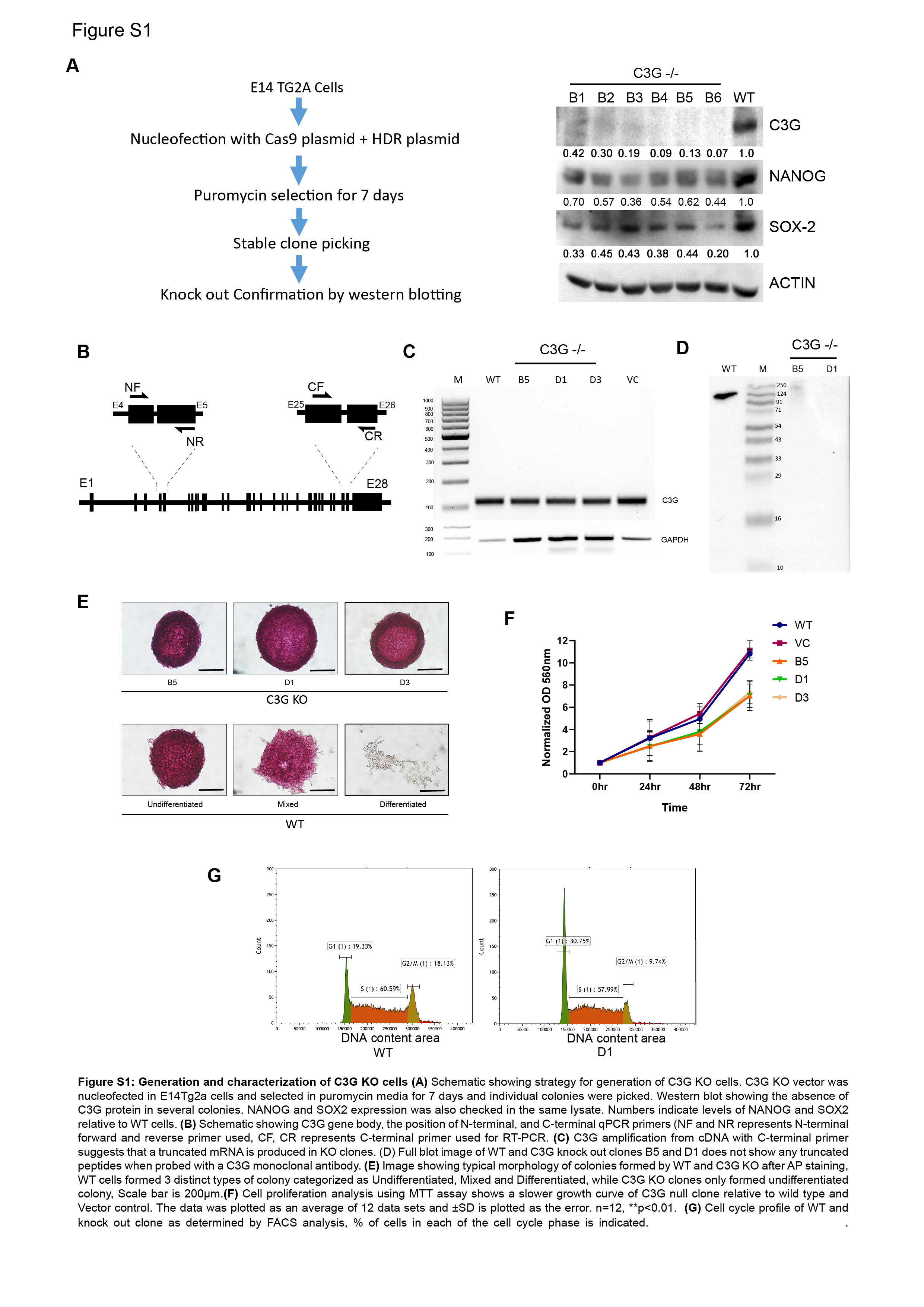

Supplement: Supplementary file 2 — (JPEG 1.55 mb) [file 12015_2021_10136_MOESM2_ESM.jpg]

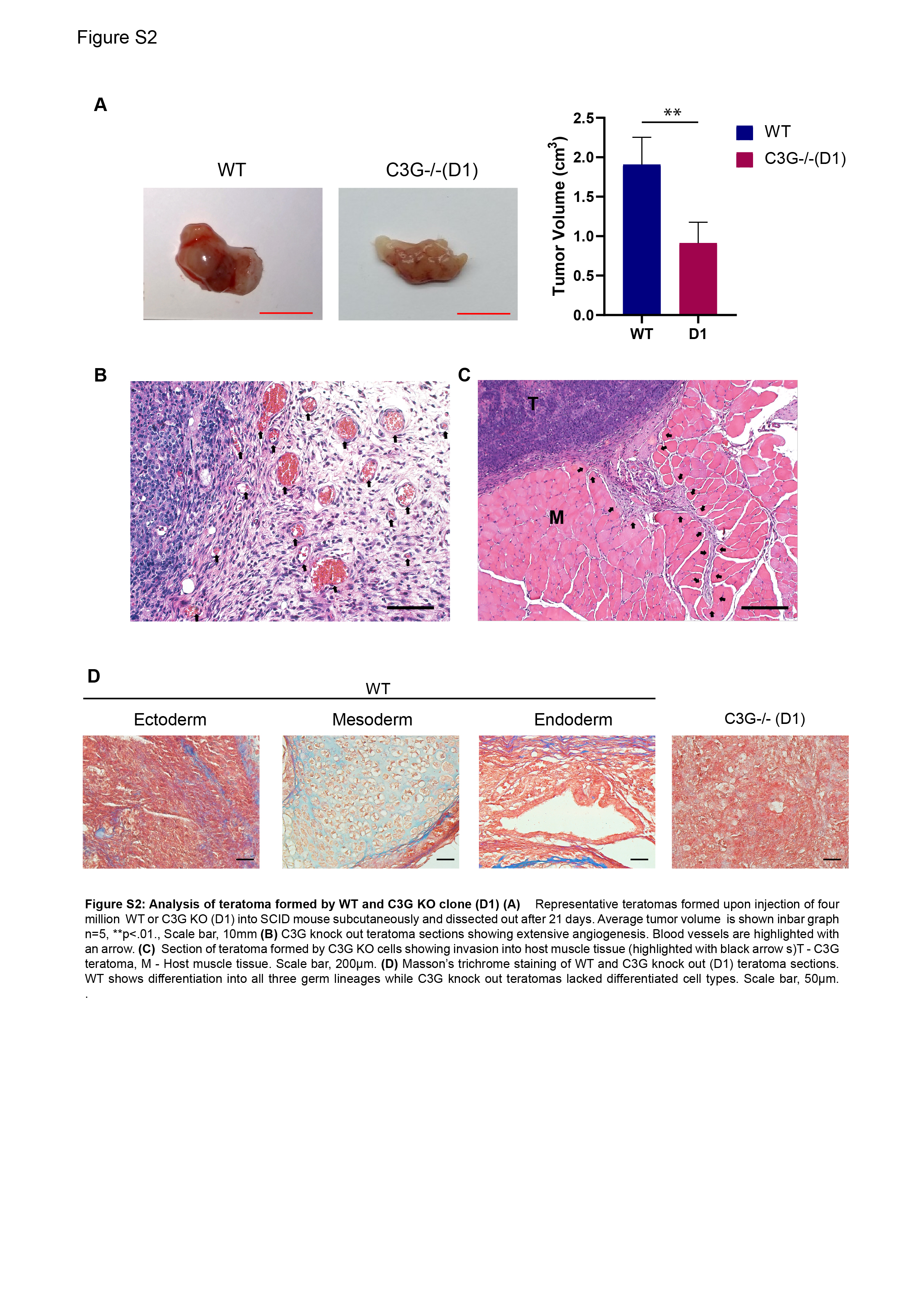

Supplement: Supplementary file 3 — (JPEG 3.54 mb) [file 12015_2021_10136_MOESM3_ESM.jpg]

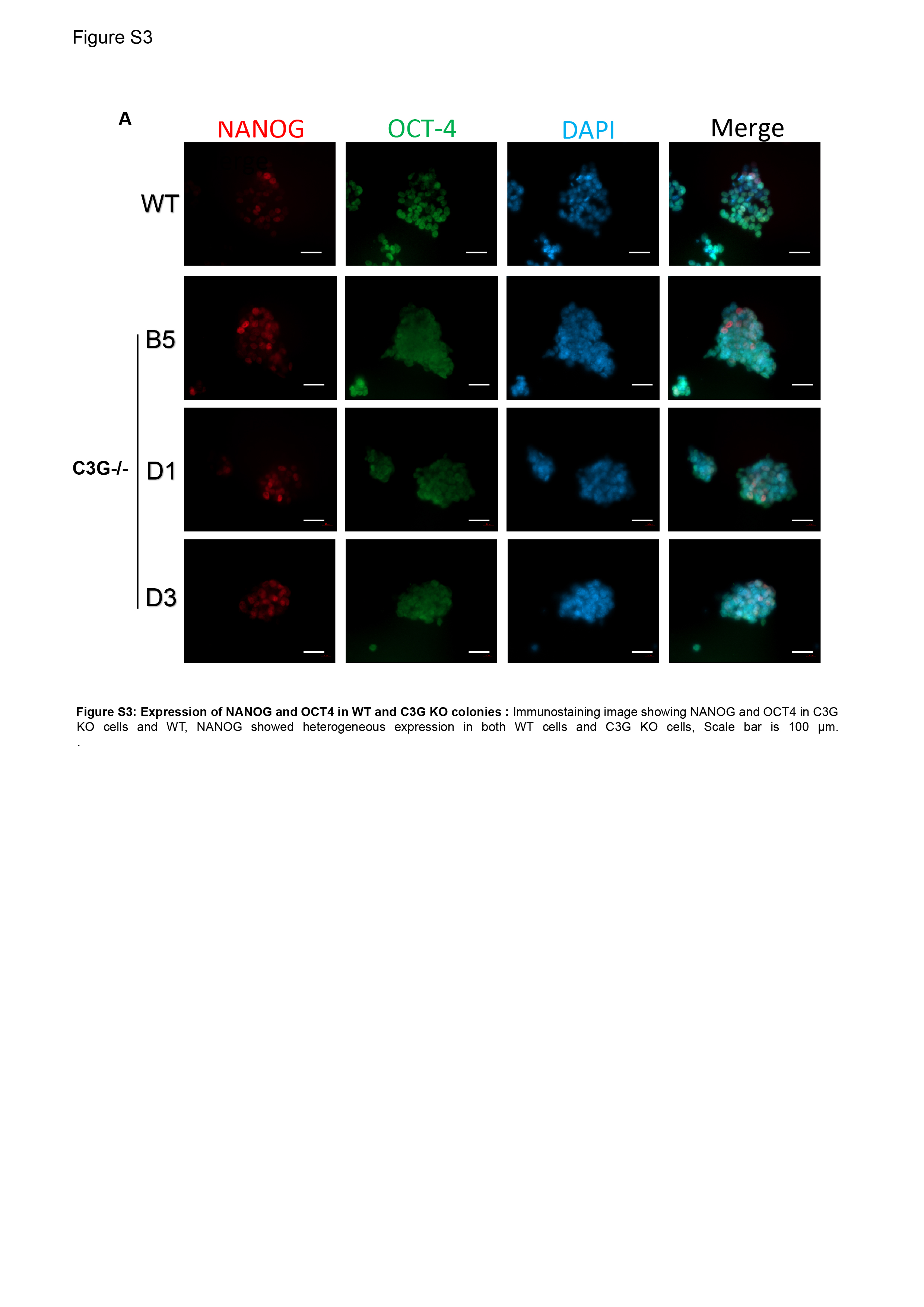

Supplement: Supplementary file 4 — (JPEG 746 kb) [file 12015_2021_10136_MOESM4_ESM.jpg]

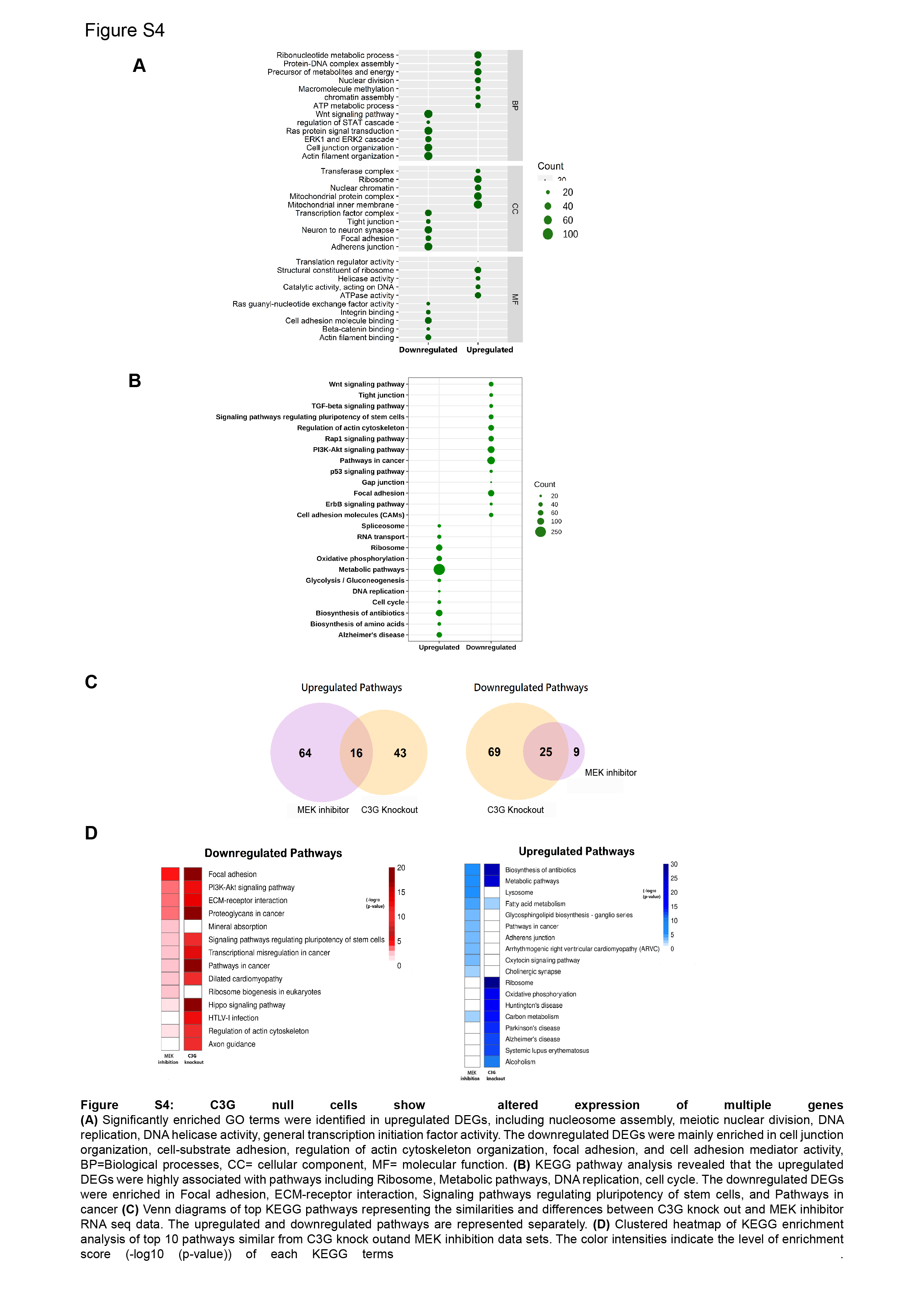

Supplement: Supplementary file 5 — (JPEG 1.43 mb) [file 12015_2021_10136_MOESM5_ESM.jpg]

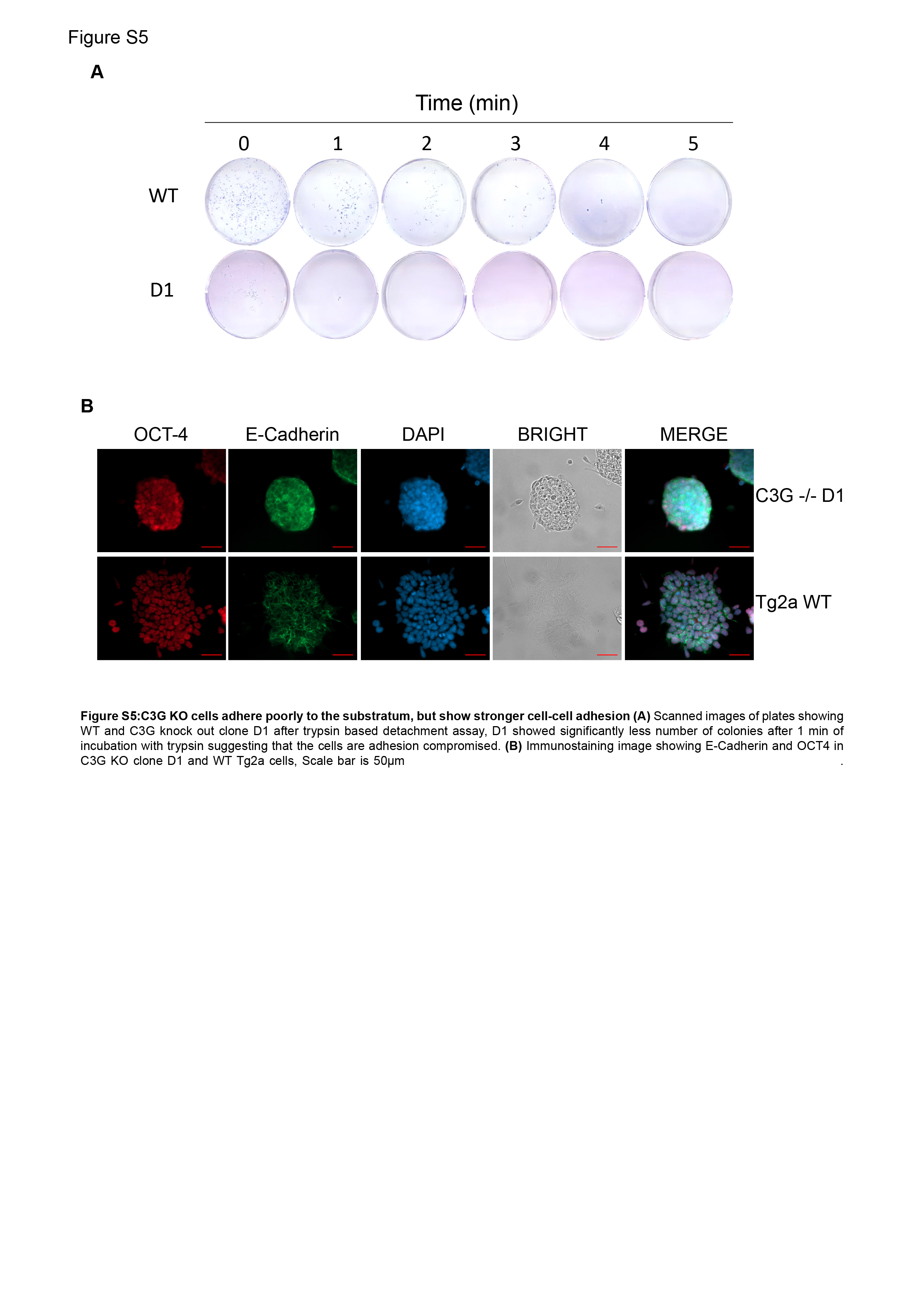

Supplement: Supplementary file 6 — (JPEG 1.12 mb) [file 12015_2021_10136_MOESM6_ESM.jpg]
